# Extremely high spatiotemporal resolution microscopy for live cell imaging by single photon counting, noise elimination, and a novel restoration algorithm based on probability calculation

**DOI:** 10.3389/fcell.2024.1324906

**Published:** 2024-06-24

**Authors:** Daisuke Miyashiro, Takuro Tojima, Akihiko Nakano

**Affiliations:** Live Cell Super-Resolution Imaging Research Team, RIKEN Center for Advanced Photonics, Wako, Saitama, Japan

**Keywords:** high-speed and super-resolution live imaging, single photon counting, noise elimination, novel restoration algorithm, probability calculation, Golgi apparatus, TGN, clathrin vesicles

## Abstract

Optical microscopy is essential for direct observation of dynamic phenomena in living cells. According to the classic optical theories, the images obtained through light microscopes are blurred for about half the wavelength of light, and therefore small structures below this “diffraction limit” were thought unresolvable by conventional optical microscopy. In reality, accurately obtained optical images contain complete information about the observed objects. Temporal resolution is also important for the observation of dynamic phenomena. A challenge exists here to overcome the trade-off between the time required for measurement and the accuracy of the measurement. The present paper describes a concrete methodology for reconstructing the structure of an observed object, based on the information contained in the image obtained by optical microscopy. It is realized by accurate single photon counting, complete noise elimination, and a novel restoration algorithm based on probability calculation. This method has been implemented in the Super-resolution Confocal Live Imaging Microscopy (SCLIM) we developed. The new system named SCLIM2M achieves unprecedented high spatiotemporal resolution. We have succeeded in capturing sub-diffraction-limit structures with millisecond-level dynamics of organelles and vesicles in living cells, which were never observed by conventional optical microscopy. Actual examples of the high-speed and high-resolution 4D observation of living cells are presented.

## Introduction

Optical microscopy is a powerful tool for observing dynamic phenomena in living cells. Electron microscopy has made enormous contribution for revealing details of small structures within cells, but understanding their dynamic aspects requires analysis of living cells. The discovery of green fluorescent protein in the late 20th century by Shimomura and others (Shimomura et al., 1962; Chalfie et al., 1994; Tsien, 1998; Shimomura, 2005) brought about a revolution in cell biology and opened a new era of live imaging. Since then, the progress in microscopic technology has been rigorously pursued.

One big issue is the improvement of spatial resolution of optical microscopy. It was long considered to be limited by the wavelength of the light, according to the classic theories of Abbe and Rayleigh (Abbe, 1873; Novotny and Hecht, 2012). Techniques to overcome this diffraction limit, called super-resolution microscopy, have been developed (Hell and Wichmann, 1994; Betzig et al., 2006; Rust et al., 2006) and are already in wide use for life science research. However, they are not necessarily suitable for observing dynamic behaviors in living cells, because these methods realize high spatial resolution often by sacrificing temporal resolution.

The classic theories of diffraction limit considered that the information obtained from the image was partly cut off by the lens and was therefore not sufficient to reconstruct the original structure. Mathematically, there is a method to reconstruct the original image from the diffracted light information, which is called deconvolution. However, due to the cutoff of the image information, conventional deconvolution has been regarded as an approximation method and thought to be insufficient to achieve super-resolution reconstruction. According to the sampling theorems of Shannon and Harris (Shannon, 1949; Harris, 1964), it should be possible to calculate and restore the original structure of the observed object at much higher resolution if a microscopic image is obtained with sufficient accuracy.

The restorability of spatial resolution essentially stems from the fact that a one-to-one correspondence is always established between the image after transmission through the optics and the original one. It is true even if the frequency band carrying the information is finite, provided that the field of view is finite and the amount of information is infinite. There is an upper limit of the spatial frequency that can be directly transmitted by the optical system, but it is extrapolatable under certain conditions based on appropriate probability calculations. Such a method has not been realized so far, because the data accuracy obtained by conventional microscopy was not high enough to execute such calculation. There were previous attempts to improve resolution by computational restoration based on the ideas similar to the present study (for example, see Agard et al., 1989). The problem was that the methods were based on point estimates and thus could not be adequately evaluated in terms of reliability. Therefore, in practical situations, the improvement of resolution has been limited to a small range and the reliability argument was obviated.

Consideration of temporal resolution is also critical for live imaging of small objects. From a thermodynamic perspective assuming random Brownian motion, the smaller the object is, the faster it moves. There is a trade-off between the measurement time and the measurement accuracy. Therefore, the observation of high-accuracy image information at high speed is always a challenge. Nevertheless, we have been eager to visualize dynamic phenomena of small intracellular structures such as organelles and vesicles in living cells. Key is to increase the absolute amounts of signals.

Here we show that, by collecting a large amount of information at high accuracy from a new microscopic system we developed, and by performing high-dimensional interval estimation in the image space, we are now able to perform a rigorous restoration of original structures and maximize spatial resolution based on reliability indices. Furthermore, we have enabled the inclusion of the time axis in the image space to be restored. Thus, we can now describe both spatial and temporal resolutions in a unified manner, so that the spatiotemporal resolution is optimized for super-resolution live imaging.

The novel high-speed and high-precision microscopy system (named SCLIM2M) we have developed and the novel method of information recovery are described below. The effectiveness of the method is shown based on the tests of its resolution and the results of actual live cell observations. The theoretical potential of this methodology will also be discussed.

## Materials and methods

### Equipment configuration

**FIGURE 1:**
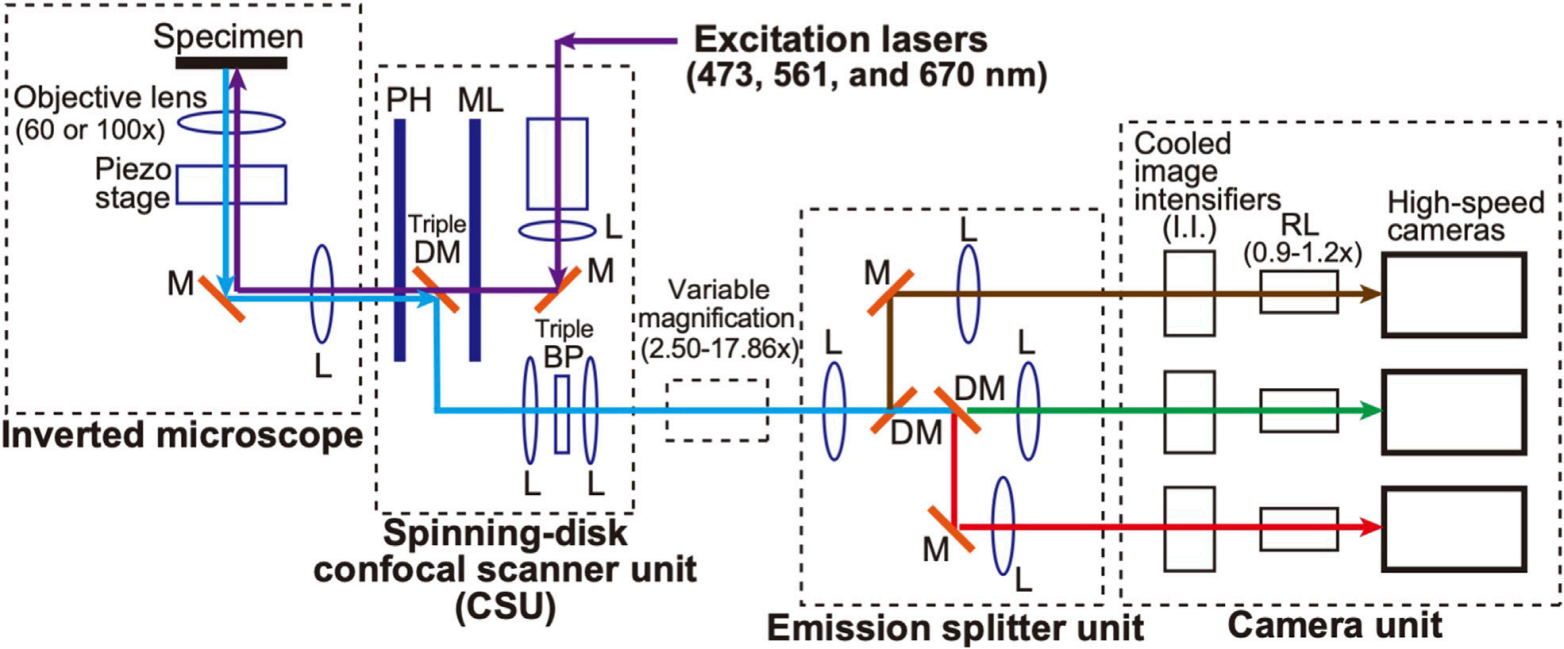
Diagram of the SCLIM2M system. An inverted microscope is equipped with a spinning‐disk confocal scanner unit (CSU), three excitation lasers (473 nm for green, 561 nm for red, and 670 nm for infrared fluorescence), a custom‐made emission splitter unit, and a camera unit containing three high‐speed cameras and three image intensifiers (I.I.) with custom‐made cooling systems (not shown in this figure). A piezo stage is placed under the objective lens (100x or 60x) to drive fast z‐scan. The confocal scanner unit contains a pinhole array disk (PH), a microlens array disk (ML), a custom-made triple-dichroic mirror (DM), a custom-made multi-bandpass filter (BP), and additional apertures for noise reduction (not shown in this figure). The emission splitter unit combines dichroic mirrors (DM), band-pass filters (not shown in this figure), relay optics, and additional magnification optics (2.50‐17.86x). The overall magnification of the optical system can be selected in the range of 150‐1786x. The magnification of the relay lens (RL) between I.I. and the high-speed camera can be fine-tuned (0.9‐1.2x) to optimize the single photon counting process. The purple arrows indicate the light paths for the excitation lasers. The green, red, brown, and light‐blue arrows indicate green, red, infrared, and their mixed multiwavelength fluorescence emitted by the specimen, respectively. L, lens. M, mirror.

A schematic of the microscopic system used in this study is shown in Figure 1. A spinning-disk confocal scanner (CSU) (Yokogawa Electric, CSU-X1) (see Tanaami et al., 2002) and an inverted microscope (Nikon, ECLIPSE Ti2) are used as a basis to which a home-made optical system is added. This configuration is basically inherited from our original SCLIM (super-resolution confocal live imaging microscopy) model (SCLIM1) (Kurokawa et al., 2013; Kurokawa et al., 2019), but with many new features as described below, we named the present system SCLIM2M (second generation Miyashiro model) (see also Tojima et al., 2023, for SCLIM2K model).

Weak autofluorescence is generated when a laser beam, the excitation source, passes over the pinhole disk of CSU. The intensity of this autofluorescence is not problematic in conventional observation, but at the level of accuracy in this study, it is the largest source of noise generated by the optical system. To reduce it, the optical filter was optimized and an additional aperture was added in the CSU unit.

Three objective lenses were used (Nikon/TIRF 60XC oil, NA 1.49; TIRF100XC oil, NA 1.49; Lambda S 100XC silicone, NA 1.35), depending on the object of observation. Three lasers with emission at 473 nm (Kyocera SOC, J050BS-1A/CW, DPSS, 50 mW/), 561 nm (Kyocera SOC, J050YS-1A/CW, DPSS, 50 mW), and 670 nm (MPB Communications, VFL-P-200-670-OEM1-B1-F4M/CW, 200 mW) were used as the light sources for excitation. A custom-made emission splitter unit was used for simultaneous observation at three wavelengths (ex473/em510, ex561/em610, and ex670/em690). Multi-pass bandpass filters and dichroic mirrors were custom-made, and emission filters were selected from commercial products (Semrock, bandpass filter series) for each sample.

For the light measurement part, three pairs of an image intensifier (I.I.) cooled at −25°C (Hamamatsu Photonics, C9016-32, a model of two microchannel plates (MCP) with a custom-made Peltier cooling system) and a high-speed camera (Photron, FASTCAM Mini WX, 2048 × 2048 pixels, max 1,080 frames/s at the full frame) were used. A custom-made piezo stage (Mess-Tek, MS-RK30LC with modifications) was used for controlling the z-axis position of the objective lens. A custom-made software was used to control the entire apparatus.

### Data acquisition and analysis

**FIGURE 2:**
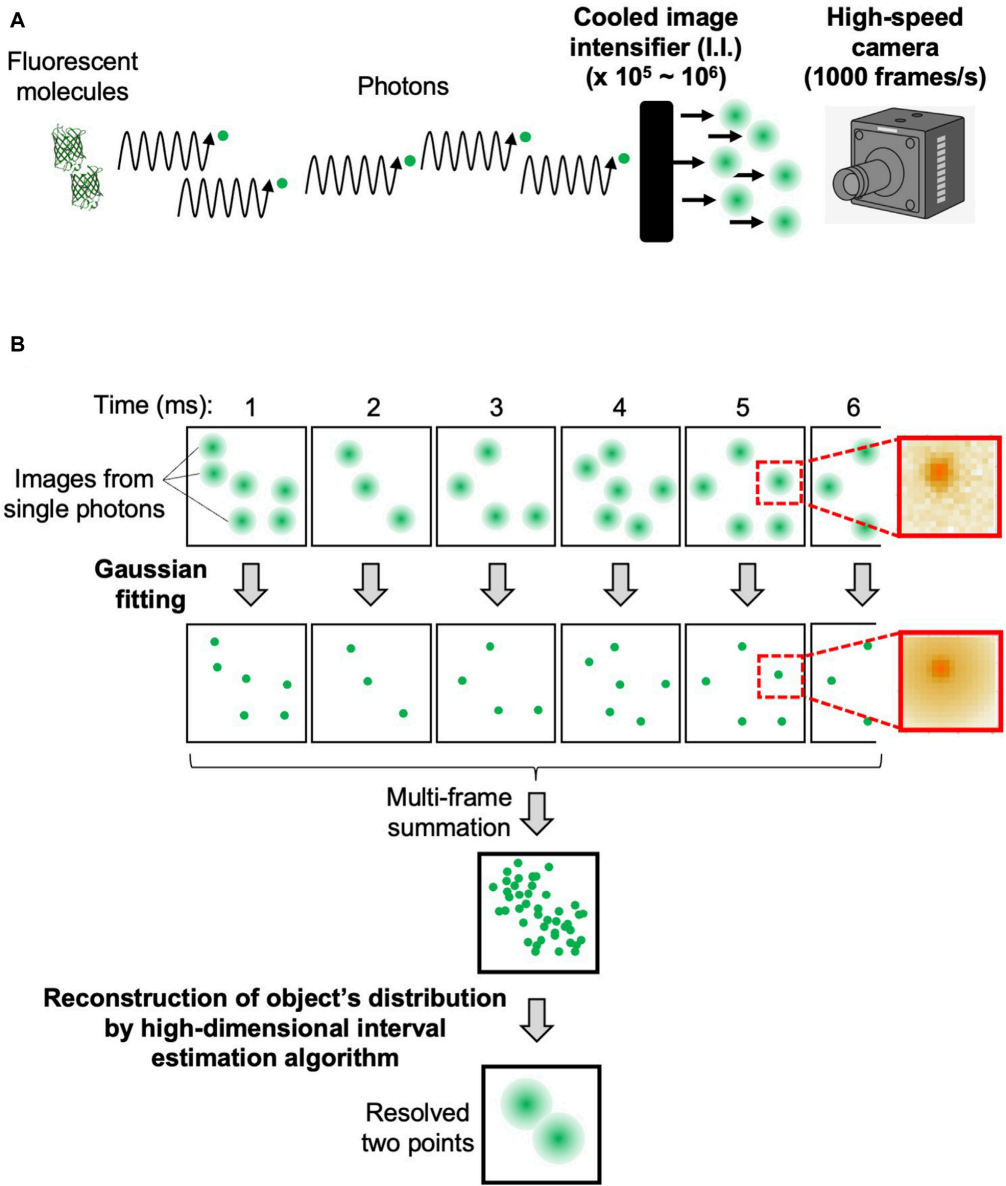
Overview of data acquisition and analysis. The flow of data acquisition and analysis procedures of SCLIM2M to achieve high spatiotemporal resolution. **(A)** Photons emitted by fluorescent molecules are transmitted through the optical system, amplified by the cooled image intensifier (I.I.), and recorded by the high‐speed camera. **(B)** The first row shows the images of each frame, and on the right is an example of the actual single‐photon‐derived image on the camera. The second row shows the positions of the photons determined by Gaussian fitting, and on the right is the single‐photon image resulting from the fitting. The lower two images show the distribution of the detected photons in 4D and the positions of the original two fluorescent molecules resolved as a result of the reconstruction calculation. In this figure, two fluorescent molecules (as in panel A) are supposed to emit photons in a stochastic way, and single photon counting with accurate determination of x‐, y‐, z‐, and t‐coordinates (panel B) leads to the reconstruction of original positions of fluorescent molecules with an assured probability calculated.

The flow of data acquisition and analysis procedures are shown in Figure 2. The confocal microscopic signals separated into three-color channels are amplified 10^5^–10^6^-fold by cooled I.I. and recorded as pixel data by the high-speed cameras (Figure 2A). By adjusting the magnification of the optics and the frame rate of the camera that suit the sample under observation (see Supplementary Appendix S2), we can obtain images in which clusters of signals are clearly identified as derived from single photons (Figure 2B, first row). On these images, the single photon counting image processing (see Supplementary Appendix S2) is applied to obtain a data set of single photons with accurate 4-dimensional (4D) coordinates (x, y, z, and t) (Figure 2B, second row). For this process, we developed our own software. The principle is based on Gaussian fitting to accurately determine the center position (x- and y-coordinates) of each signal cluster. Since the images are taken at different z-positions (by the piezo actuator) at consecutive time points (for example, every 1 ms), the data set contains accurate z- and t-coordinate values (Figure 2B, see lower image: multi-frame summation). It should be noted here that the noises that arose in the cameras are almost completely removed by this process (Supplementary Appendix S2; Supplementary Figure S1).

Then reconstruction calculation is performed to build the image of the original object from the density of detected photons in 4D (Figure 2B, see lower image: resolved two points). The calculation method is detailed in Supplementary Appendix S3. A GPGPU computer (NVIDIA, NVIDIA V100) and the original program we developed (CUDA-C) were used for this calculation. The resulting images can be output in several formats. Our routine is to first obtain moving images of a multi-colored 3-dimensional (3D) opacity display (by Volocity, PerkinElmer) that can be interpreted in the same way as images of conventional fluorescence microscopy. The intermediate data with probability calculated are also stored for further detailed analyses (by Mathematica, Wolfram). The numerical performance of the microscopic system SCLIM2M is summarized in Table 1.

**TABLE 1:** Image acquisition details of SCLIM2M.

Item	Specification	Range of use
Excitation light power	473 nm: Max. 24 W/cm^2^	0.01%–100%
	561 nm: Max. 33 W/cm^2^	
	670 nm: Max. 100 W/cm^2^	
Number of pixels	Max. 2000 × 2000 pixels	1,500 × 1,500–1,000 × 1,000 pixels
Size of pixels	Min. 5.6 nm	5.6–40 nm
Field of view	Max. 60 × 60 μm^2^	5.6 × 5.6–60 × 60 μm^2^
Flame rate of xy-plane	1,000 frames/s	
Data storage	Max. 32 GB ([xy-pixels] × [z-slice] × [time points])	
Continuous imaging period (triangle wave)	Max. 20 s (400 volumes)	2.5–20 s (50–400 volumes)
Time-lapse imaging period (saw wave)	Determined by the time-lapse interval (max. 400 volumes)	

### Single photon counting

Among various methods of single photon counting on image processing, we employed a computationally inexpensive method in this study. Its accuracy is validated as described in Supplementary Appendix S2.

First, local maximum points are extracted from the entire field of view of the camera in the range of 9 pixels of 3 × 3. If the image in this area is derived from a single photon, the ratios of the intensity values of the center point to those of the surrounding 8 pixels should fall within a certain range (Supplementary Figure S1B). By measuring and evaluating the background noises of the camera in advance, we can also create a judgment condition that eliminates noise effects. The position of the center of gravity of the image and the time of observation are recorded as point data with single coordinates (x, y, and t). They are compared to the 3D scanning mode information to add z values and obtain a collection of point data with accurate 4D coordinates (x, y, z, and t).

### High-dimensional interval estimation algorithm

In general, estimation methods can be classified into point estimation and interval estimation. The conventional deconvolution method corresponds to point estimation, which selects a single most promising candidate image based on certain statistical criteria. On the other hand, interval estimation, on which the present method is based, handles a set of images within a certain confidence level. Although the space representing the entire candidate images is very high-dimensional and computationally expensive, as shown in the Supplementary Appendix S3, we were able to obtain practical results by computing images corresponding to the expected values in the image space based on assumptions generally made in fluorescence microscopy.

### 3D scanning mode

**FIGURE 3:**
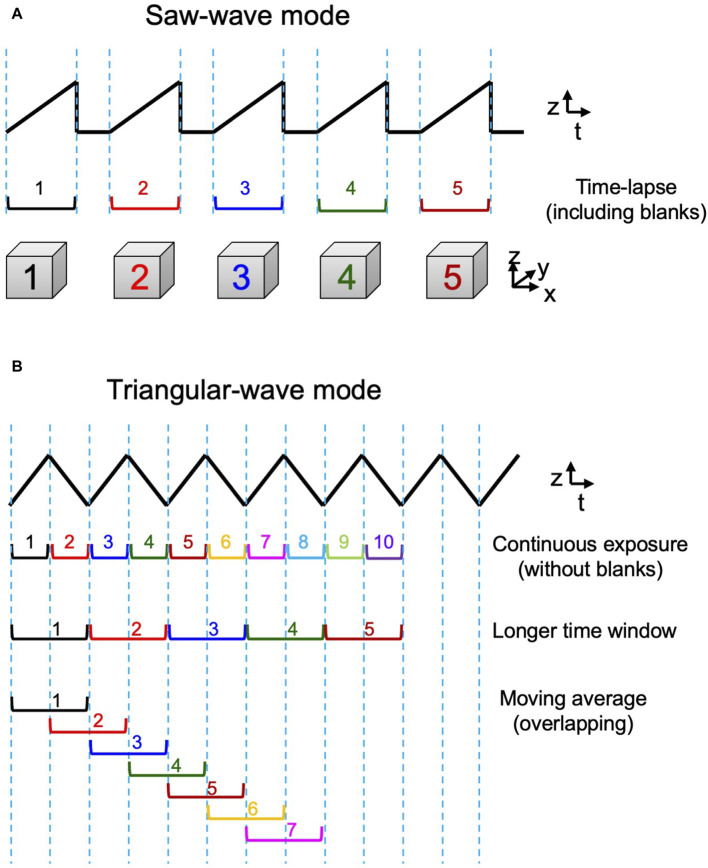
3D scan mode. Two modes of z‐axis scanning. **(A)** The saw‐wave scanning mode employs linear scanning of the object lens in a single direction separated by a blank time to return to the beginning position. **(B)** The triangular‐wave scanning mode takes up-and-down two‐way scanning and is operated without any blank time. 3D data obtained by both up and down directions can be used consecutively (1st row) or pairs of up and down sequences can be combined to give data of a longer time window (2nd row). Furthermore, such longer window data can be also used in an overlapping way to achieve moving average 4D presentation (3rd row). See examples in Figure 7B and C.

By the custom-made piezo actuator controller, the system can choose either a conventional saw-wave scanning mode or a triangular-wave scanning mode using the dynamic characteristics of the piezo actuator (Figure 3). For its control, the waveform input to the piezo was shaped to match the actual behavior of the piezo stage. The movements of the piezo and the objective lens under actual measurement conditions were carefully tested to ensure accuracy (Supplementary Figure S3). An aluminum block with the mass and shape as almost the same as the objective lens was installed on the piezo stage and their movements were monitored with an external capacitance sensor (Mess-Tek, TRA316-20). The measurements were performed for 5-axis (x, y, z, θx, and θy) not only to confirm the accuracy in the z-direction but also to estimate crosstalk in the non-scanning direction. The effect of the surface tension force exerted through the oil between the sample and the objective lens at high speed was also confirmed negligible under practical conditions (Supplementary Figure S3).

The favorable behavior of the lens as shown in Supplementary Figure S3 could be disturbed at higher driving frequencies. In this study, the triangular-wave mode was operated at 10 Hz and the saw-wave mode was used with a static duration of 50 ms or longer, which were confirmed safe for stable operation.

### Measurement of point spread function (PSF)

Fluorescent beads (Micromod Partikeltechnologie, sicastar-redF/greenF, made of silica, particle size 25 nm in diameter) were adhered to the surface of a glass coverslip (Matsunami) to measure PSF. The beads were suspended at an appropriate concentration in 1 M NaCl solution and poured between two coverslips. The space between the two coverslips was about 3 µm thick due to the surface tension.

### Sample preparation

DNA origami (Matsubara et al., 2023) was kindly provided by Mitsuhiro Iwaki (National Institute of Information and Communications Technology). For microscopic observation, two glass coverslips were put together with double-sided transparent adhesive tape as a spacer to make a chamber. To the chamber, 10 µL of biotinylated BSA (bovine serum albumin) (10 mg/mL) was added, let stand for 4 min, and washed by 30 μL TE buffer (10 mM Tris-HCl and 1 mM EDTA, pH 8.0). Then 5 μL of neutravidin (5 mg/mL, Thermo Fisher) was added, let stand for 4 min, and washed by TE buffer. Finally, 10 µL of the DNA origami suspension in TE buffer was poured into the chamber, let stand for 4 min, and washed by TE buffer again. Biotinylated BSA was prepared by incubating 1 mL of 10 mg/mL BSA (Sigma-Aldrich) with 1 mg of biotin-(AC_5_)_2_-OSu (Dojindo) in 10 µL DMSO for 1 h at room temperature.

Human HeLa cells (RIKEN BioResource Research Center) were maintained and seeded on glass-based dishes for microscopic observations as described previously (Tojima et al., 2023). For imaging of actin filaments and microtubules, cells were fixed with a fixation buffer (80 mM Na-PIPES, pH 6.9, 1 mM MgCl_2_, 1 mM EGTA, 3% sucrose, 0.1% glutaraldehyde, and 4% formaldehyde) for 30 min at 37°C, permeabilized and blocked with 0.2% Triton X-100 and 10% goat serum for 60 min, and then incubated with Alexa568-conjugated anti-β tubulin antibody (1:300, Abcam, EPR16774) and Alexa488-conjugated phalloidin (1:200, Thermo Fisher, A12379) overnight at 4°C. The cells were then mounted with SeeDB2S solution (Ke et al., 2018). For live imaging of the Golgi apparatus, cells were transfected with expression plasmids encoding mCherry-ManII (aa1-112 of mouse mannosidase 2α1, Addgene, 54150), mEmerald-TGN46 (Addgene, 54279), and iRFP713-ST (aa1-45 of human β-galactoside α-2,6-sialyltransferase 1) (Tojima et al., 2024), using Lipofectamine 3000 reagent (Thermo Fisher) according to the manufacturer’s protocol.

To visualize clathrin dynamics at the *trans*-Golgi network (TGN) of the budding yeast *Saccharomyces cerevisiae*, we used the strain YSY1 (YPH499 *ADE2::pRS402 CLC1-GFP(S65T)::HIS3MX6*) and the low-copy plasmid pRS316-ADH1p-SEC7-tagRFP (Tojima et al., 2019). Here, Clc1 is the clathrin light chain, and Sec7 is used as a TGN marker. For microscopic observation, the yeast cells were grown in selective medium (0.67% yeast nitrogen base without amino acids and 2% glucose) with appropriate supplements. The cells were harvested at early-to mid-logarithmic phase and then seeded onto glass coverslips coated with concanavalin A.

## Results

The methodology of this study, which has been implemented in the new microscopic system SCLIM2M, is based on two aspects: high-precision optical measurement and precise handling of the measured information. Mathematical description is given in Appendices.

Measurements are carried out by optical image acquisition with a custom-made spinning disk (CSU) system and high-speed cameras (1,000 frames/s), which fulfills high temporal resolution. Single photon counting at this high speed has been made possible by a high-magnitude signal amplification with custom-made cooled I.I. devices and by a computational image processing including complete removal of camera-derived noises. It enables us to obtain data of much higher S/N (signal to noise ratio) than conventional microscopic observation (see Materials and Methods and Figures 1–3).

A novel method has also been developed to reconstruct the structure of the observed object using the massive amount of information (10^6^ times larger than conventional methods). It is based on the sampling theorems of Shannon and Harris (Shannon, 1949; Harris, 1964), which indicate that the images before and after optical measurement are in one-to-one correspondence even if the transmitted spatial frequencies are restricted. The original images can be reconstructed by extrapolation of the observed information with appropriate probability calculation (see Supplementary Appendix S3). Thus, we can rebuild the nano-scale structure of the original object and discuss its reliability based on quantitative statistics.

Now we describe the tests to validate the high spatial resolution of SCLIM2M and the results of actual live imaging to demonstrate the practical potential of SCLIM2M in visualizing organellar and vesicular dynamics at high spatiotemporal resolution.

### Demonstration of spatial resolution using DNA origami

**FIGURE 4:**
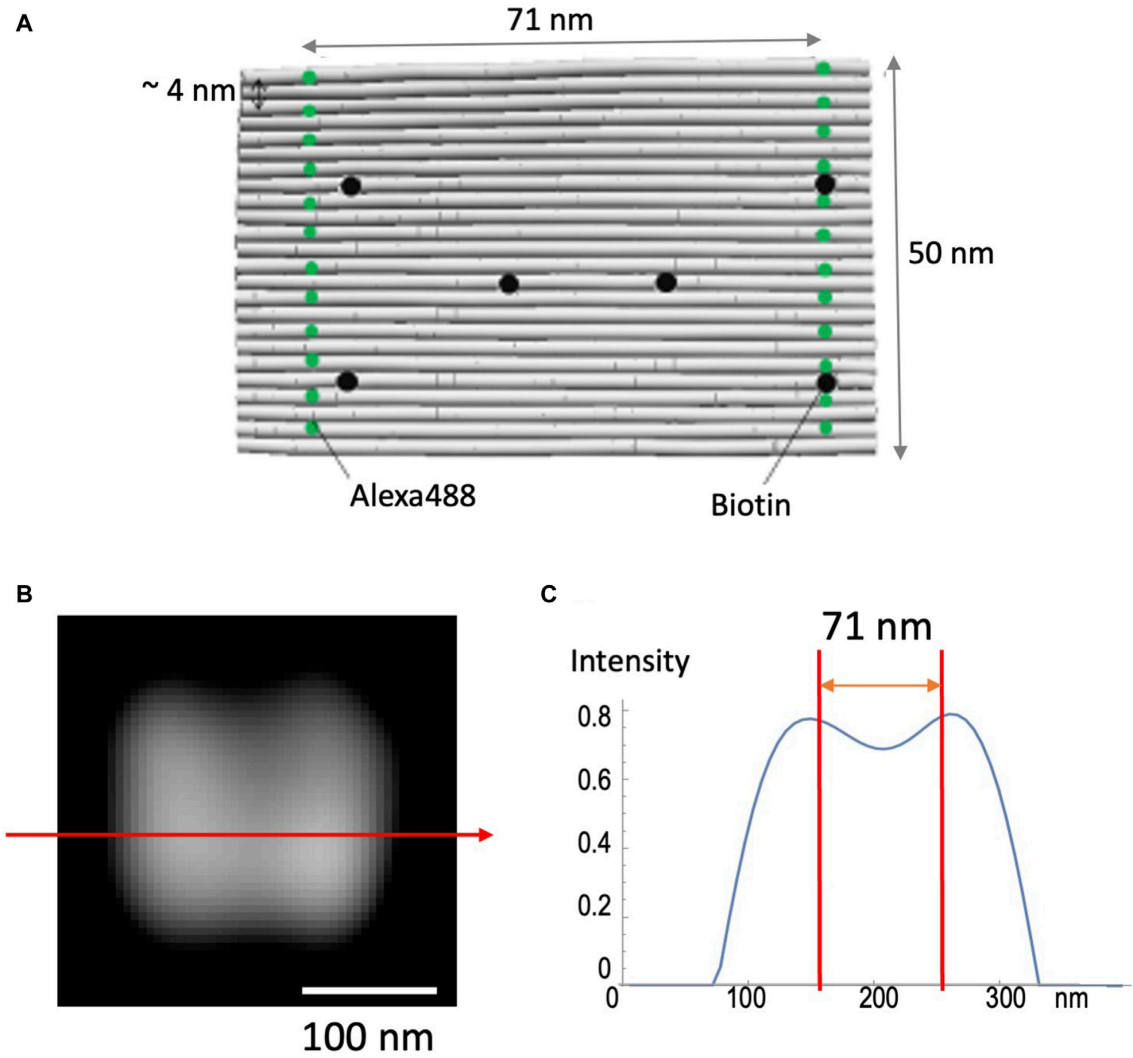
Observation of nano-ruler. **(A)** A 24-helix bundle DNA origami sheet was used as a nanometer-accurate ruler, in which fluorescent dyes (Alexa488) are aligned along parallel lines separated by 71 nm. Biotin is designed to fix the ruler to glass coverslips. **(B)** A field of view as measured by SCLIM2M and processed for single photon counting and image reconstruction. Bar, 100 nm. **(C)** The line profile along the red arrow in **(B)**. The intensity is in arbitrary units. Two red lines indicate 71-nm distance.

DNA origami (see Materials and Methods) was used to test the validity of the method to achieve high spatial resolution (Figure 4). This nano-ruler has fluorescent dyes aligned along parallel lines separated by 71 nm (Figure 4A). Figures 4B, C shows that SCLIM2M indeed resolves this distance in 2D space, enabling the 71-nm accuracy far beyond the diffraction limit. It also confirms the methodological consistency of quantitative relationship between the amount of signals and the resolution. In the case of the measurement shown in Figure 4B, about 8000 photons were collected as valid signals.

### Demonstration of 3D resolution using fixed cells

**FIGURE 5:**
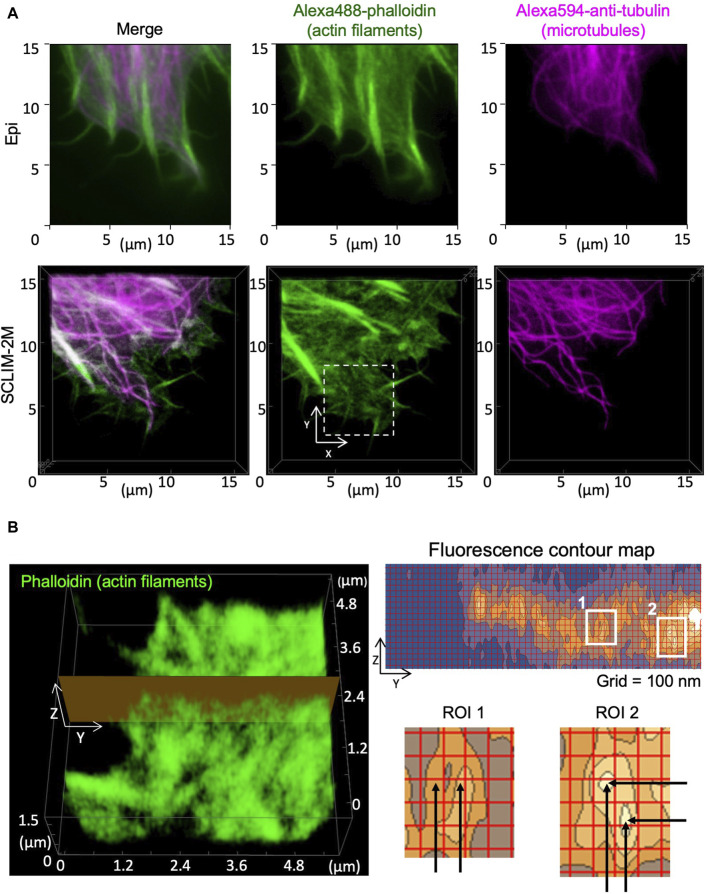
Observation of cytoskeletons in fixed HeLa cells. Actin filaments and microtubules in chemically fixed HeLa cells were visualized by phalloidin (green) and anti-tubulin antibody (magenta) staining. **(A)** Epifluorescence images (upper panels) and SCLIM2M images (lower panels) of the peripheral regions of the cells. Right, center, and left panels show microtubules, actin filaments, and their merged images, respectively. **(B)** Left panel shows a zoom‐up image of actin filaments in the white dashed box drawn in **(A)**. Right panel (grid size 100 nm) shows a fluorescence contour map of a zy section drawn in the left panel. Spatial resolution of signals is approximately 100 nm in the y‐direction and 150 nm in the z‐direction, respectively, as shown in enlarged regions of interest (ROI1 and ROI2, see the distance between black arrows).

Spatial resolution in 3D was tested by observing cytoskeletons in fixed HeLa cells (Figure 5). Images of actin filaments and microtubules obtained by conventional epifluorescence microscopy (epi) (Figure 5A. upper panels) and by SCLIM2M (Figure 5A, lower panels) were compared. Higher spatial resolution of the latter was clearly seen. Because the accuracy in z-coordinates depends on piezo scanning of the objective lens, it needs to be verified experimentally (see Supplementary Figure S3). As shown in Figure 5B, apparent spatial resolutions for actin filaments were approximately 100 nm in y-axis and 150 nm in z-axis. It indicates that restoration beyond the transmission band of the optical system was successful (see Supplementary Appendix S3). It should also be noted that the validity of SCLIM2M was demonstrated under a non-uniform refractive index environment, where local variations may exist in PSF (Supplementary Appendix S4).

### 4D observation of the Golgi apparatus in living HeLa cells and demonstration of dynamics of small structures in real time

Next we extended our imaging analysis by SCLIM2M to the Golgi apparatus in living cells. The Golgi apparatus in most eukaryotic cells consists of stacked layers of several flattened bladders called cisternae as manifested by electron microscopy. In mammalian cells, such stacks of the Golgi are often connected with each other and form large ribbon-like structures, which are hard to resolve by conventional light microscopy (Nakano, 2022). SCLIM2M demonstrates its prospective ability to visualize very dynamic behaviors of the Golgi apparatus in detail in living HeLa cells.

**FIGURE 6:**
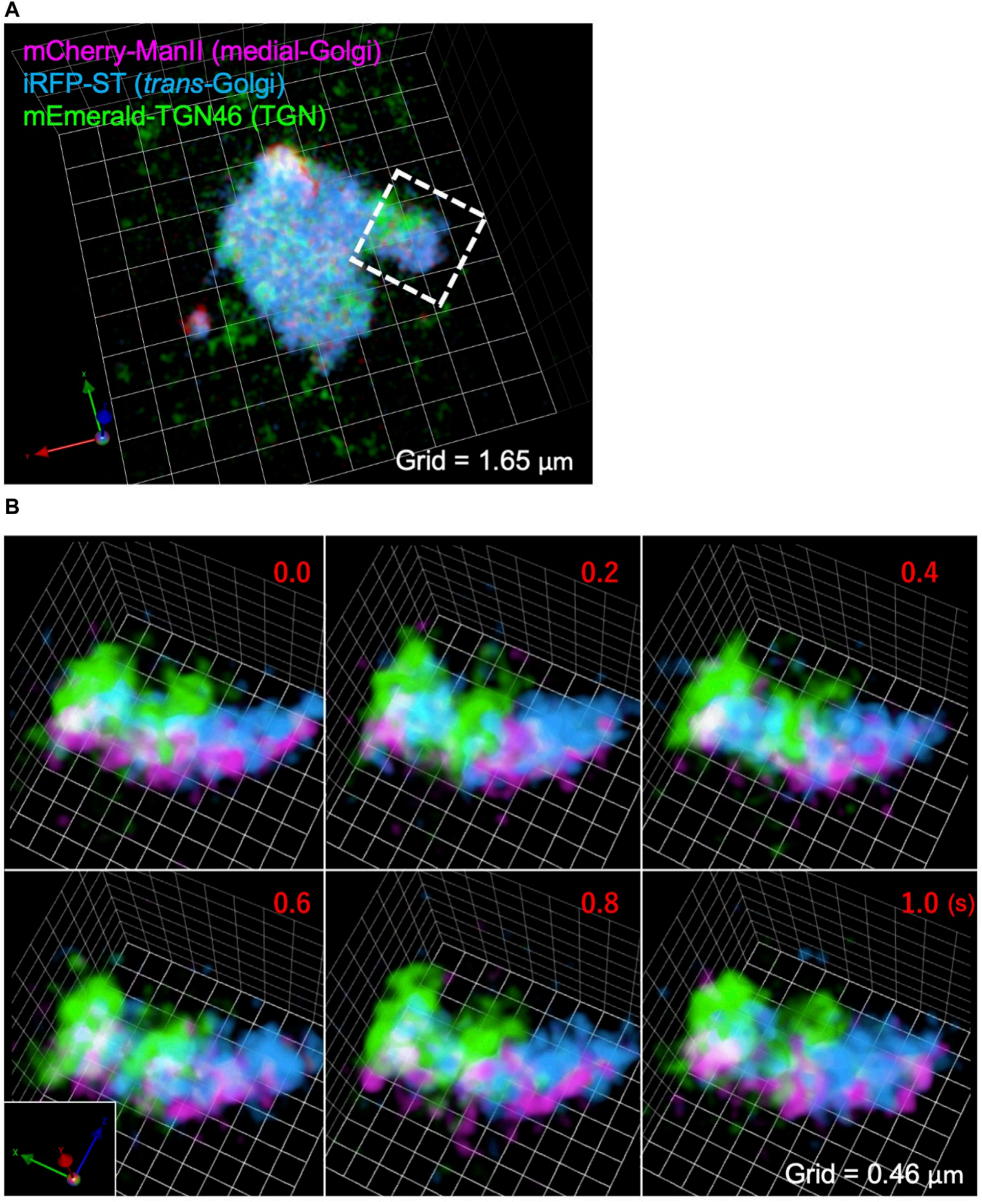
Observation of the Golgi apparatus in a living HeLa cell. **(A)** A triple-color SCLIM2M image of the Golgi apparatus in a living HeLa cell expressing mCherry-ManII (magenta, medial Golgi marker), iRFP-ST (cyan, *trans* Golgi marker), and mEmerald-TGN46 (green, TGN marker). Grid size is 1.65 µm. **(B)** Enlarged images of a portion of **(A)** (white dashed box), obtained by live imaging observation. 3D data of single photon counting measurement were subjected to the reconstruction calculation. Calculated 3D projection images are shown here. Red numbers indicate time (s) after the onset of imaging. Live images were acquired in a saw-wave mode with 75 xy planes (taken every 2 ms) moving along z-axis (66.7 nm pitch over 5 μm, total 150 ms) and 50 ms blank (see Figure 3A). Reconstructed 3D images were compiled into a movie (Supplementary Movie S1). Grid size is 0.46 µm.

Figure 6A shows a SCLIM2M image of a whole Golgi ribbon in a HeLa cell, of which three different cisternae were fluorescently labeled with mCherry (magenta, Mannosidase II as a medial Golgi marker), iRFP (cyan, Sialyl Transferase as a *trans* Golgi marker), and mEmerald (green, TGN46 as a *trans*-Golgi network marker). Figure 6B shows the enlargement of a small part of the Golgi ribbon of Figure 6A (white dashed box) as displayed as scenes of 0.2 s apart selected from a 3D movie (Supplementary Movie S1). Clear separation of the three compartments was achieved by SCLIM2M. Furthermore, unrecognized dynamic motions of the Golgi cisternae and the vesicles around them are visualized.

For this live imaging observation, the single photon counting measurement was made every 2 ms over 5 µm moving along z-axis by piezo scanning (a saw-wave mode, see Figure 3A). The stack of 75 xy planes were combined as a set of 3D volume data and subjected to the reconstruction calculation. Then calculated 4D data (including the time coordinate) were compiled into a movie (Supplementary Movie S1). From the movie, which displays projected 3D images at the rate of 5 volumes/s (real time), we can comprehend that behaviors of the Golgi cisternae and the vesicles around are very dynamic, because of the high resolution in both space and time. Such images have never been seen by preexisting optical microscopy.

### 4D observation of the *trans*-Golgi network in living yeast cells and the future possibility of tracking individual vesicles

**FIGURE 7:**
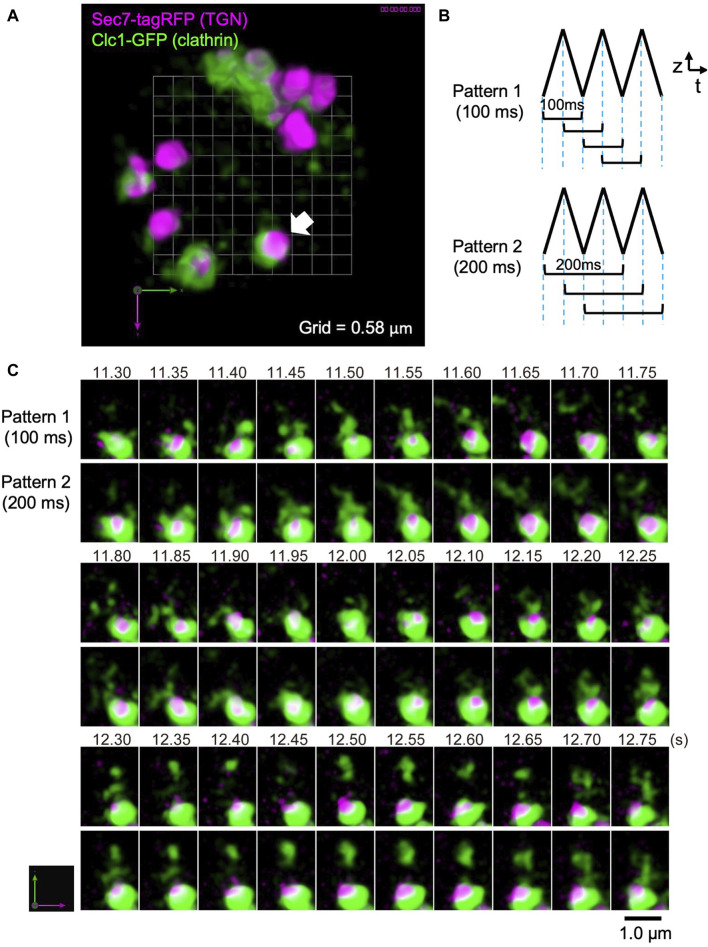
Observation of clathrin dynamics at the TGN in a living yeast cell. **(A)** A dual-color SCLIM2M image of the TGN in a living yeast cell expressing Sec7-tagRFP (magenta, TGN marker) and Clc1-GFP (green, clathrin light chain). Grid size is 0.58 µm. 3D data set (50 xy planes) of single photon counting measurement taken every 1 ms by a triangular-wave mode for 4.5-µm up and down (90 nm pitch, 50 ms each) were subjected to the reconstruction calculation and compiled into a movie (Supplementary Movie S2). **(B)** Two patterns of 3D scanning employed to compile movies. Pattern 1 is using one pair each of up-and-down (or down-and-up) data (100-ms average) to reconstruct volume data, which are displayed at consecutive 50 ms. Pattern 2 is by two pairs of up-and-down data (200-ms average), which are also displayed at consecutive 50 ms. **(C)** Sequential images over time of the TGN cisterna indicated by a white arrow in **(A)**. The numbers on top represent time (s) after the onset of imaging. The images were acquired in a triangular-wave mode and reconstructed using Pattern 1 (100-ms average) and Pattern 2 (200-ms average) of panel **(B)**. Note that the longer time averaging causes lower spatial resolution but gives us a clue to postulate directionality of movement. Bar, 1.0 µm.

As another example of live imaging analysis by SCLIM2M, we observed the *trans*-Golgi network (TGN) area of the budding yeast *S. cerevisiae* (Figure 7). TGN is known as an important sorting platform located at the *trans* side of the Golgi apparatus (Glick and Nakano, 2009; Nakano, 2022). In yeast, Sec7 protein has been shown to represent a late stage of TGN where a variety of transport carriers, such as clathrin-coated vesicles, are formed (Tojima et al., 2019, 2024). Figure 7A shows the inside of a single living yeast cell, in which Sec7 (TGN) is labeled with TagRFP (magenta) and clathrin light chain (Clc1) is tagged with GFP (green).

In this observation, a triangular-wave mode was employed for z-axis scanning (see Figure 3B). The single photon counting measurement was made every 1 ms for 4.5 µm up and down each (90 nm pitch), without any time lag. Sequences of 50 xy planes (50 ms) obtained during either up or down piezo scanning were combined and subjected to the reconstruction calculation. Figure 7B shows two patterns of moving average (see also Figure 3B). Patterns 1 and 2 collect 3D data averaging for 100 ms and 200 ms, respectively, at consecutive 50 ms. The movie obtained by Pattern 1 is shown as Supplementary Movie S2 (displayed at 20 volumes/s, i.e. real time). Figure 7C shows the behavior of the cisterna indicated by a white arrow in Figure 7A. Magenta marks TGN and green indicates clathrin assembling on TGN and running around TGN. Scenes are selected from the two movies displaying 3D structures obtained by the two scanning patterns of Figure 7B. Comparison of the two patterns with different time windows provides us with some interesting information. In the panels of Figure 7C, above the TGN cisterna (the lower large structure of ∼1 µm diameter with magenta and green), small green structures are seen which are probably clathrin-coated vesicles. Even at this high temporal resolution, tracking of individual vesicles is not easy, but by comparing the two different time averaging, we may be sometimes able to guess the directionality of movement. For example, trailing of green signals that appear to emanate from TGN at longer averaging (such as time points 11.50–11.65 s) may indicate release of vesicles, and those that appear to shrink toward TGN (for example, 11.85–11.95 s and 12.65–12.75 s) might indicate return of vesicles. Further image processing, for example by using machine learning, may help analyze their behaviors in more detail in the future.

## Discussion

We have established a novel method of super-resolution microscopy with unprecedented high speed in this study. Key technologies are massive amplification of signals by cooled I.I., image-processing-based single photon counting and noise elimination at high-speed cameras, and a probability-aware novel restoration calculation to reconstruct original structures at high precision. With the microscopic system SCLIM2M implementing this method, we now achieve spatial resolution beyond the diffraction limit, ∼70 nm in 2D and 100–150 nm in 3D, and temporal resolution as high as 1,000 frames/s in 2D and 20 volumes/s in 3D.

We would like to emphasize that the temporal resolution of our method is more than two orders of magnitude higher than the methods commonly used in live cell imaging. Such innovation is invaluable for capturing complete motion of small structures.

The spatial resolution in actual living cells, especially in the z-direction, is difficult to verify. The difficulty is due to unknown variations of refractive index within a cell as described in Supplementary Appendix S4. Instrumental reliability in the measurement along the z-axis has been confirmed as shown in Supplementary Figures S2, S3, but the reliability inside living cells is still a matter of discussion. As described in Supplementary Appendix S2, the criteria for verifying resolution are significantly different in our methodology from those in the past. Conventional restoration methods have limitations for discussing resolution because they are based on point estimation. If any known periodic structures can be observed in the cell as 3D nano-rulers, they will be helpful to examine z-directional properties.

To demonstrate the performance of SCLIM2M in resolution by comparison with other methods, we observed phalloidin-labeled actin filaments in chemically fixed HeLa cells by two pre-existing high-resolution methods belonging to image scanning microscopy (Gregor and Enderlein, 2019): spinning-disk confocal SCLIM1 with deconvolution (Kurokawa et al., 2013; Kurokawa et al., 2019) and confocal microscopy with Airyscan (Zeiss LSM980). SCLIM1 has a very high sensitivity and spatiotemporal resolution designed for high-speed live imaging and uses deconvolution by the maximum entropy method (Volocity). Zeiss’s Airyscan is chosen as an example of commercially available image scanning microscopy. The observed images are shown in Supplementary Figure S4 (SCLIM1) and Supplementary Figure S5 (Airyscan). They provide apparent resolutions from the spatial frequency (or half-width), but it should be remembered that this is different from the true resolution as shown in Figure 5, which is mathematically defined in a probability aware fashion. Nevertheless, one can easily tell the difference in “resolution” between SCLIM2M (Figure 5) and SCLIM1 (Supplementary Figure S4) and Airyscan (Supplementary Figure S5), particularly in the z-direction. The times required to obtain these images were 5 s (Figure 5), 5 s (Supplementary Figure S4), and 80 s (Supplementary Figure S5), indicating the great advantage of SCLIM microscopy in high-speed data acquisition even with SCLIM1 (see Kurokawa et al., 2013; Kurokawa et al., 2019; Tojima et al., 2019; Shimizu et al., 2021; Tojima et al., 2024). So, we next compared the performance in obtaining high-speed 3D movies between SCLIM1 and SCLIM2M. Supplementary Movie S3 is taken by SCLIM1, showing membrane dynamics around the Golgi apparatus in a living HeLa cell almost under the same condition as Figure 6 and Supplementary Movie S1. In regard to resolutions in both space and time, the superiority of SCLIM2M is obvious. As mentioned in Supplementary Appendix S3, more sensitive discussion will be required for the resolution of moving objects. It will be necessary to examine the isotropy of the observed motion on a case-by-case basis. To correlate the high-resolution 4D data of SCLIM2M with the real ultrastructure, the best way would be correlative light and electron microscopy and our efforts are now underway.

Needless to say, live cell imaging has proven extremely powerful in elucidating dynamic processes within a living cell. “Super-resolution” techniques are innovative, however their applications are restricted under moderate temporal resolution, because small structures in a living cell are often moving very rapidly. A good example is the behaviors of vesicles, important players in membrane traffic. Live cell imaging contributed very much to address big problems of trafficking such as how proteins are transported within the Golgi apparatus (Pelham and Rothman, 2000; Losev et al., 2006; Matsuura-Tokita et al., 2006; Casler et al., 2019; Kurokawa et al., 2019), but without direct observation of vesicles, ambiguity remains in precise understanding of mechanisms.

Here we present a great possibility of SCLIM2M in visualizing dynamics of organelles and vesicles in living cells. Data exemplified in this paper, on the Golgi apparatus and vesicles around in a living HeLa cell and on TGN and clathrin-coated vesicles in a living yeast cell, demonstrate that how vivid imaging in living cells can inspire our insights into what are exactly going on.

A great advantage of the methodology developed in this study is that it guarantees quantitativity in principle. The images of the object reconstructed by measurement and calculation are not only treatable like images obtained by conventional microscopy but also composed of accurate mathematical information on probability or likelihood. Such information can be used for further sophisticated analysis.

Since our methodology deals with optical measurements and subsequent analysis in general, it can also be applied to optical systems other than the spinning-disk confocal microscopy. Theoretical guidance is provided on the possibilities and limitations of application to a wide range of optical systems, and one of such optimized for this methodology will be desired to confirm its practicality.

We are not satisfied yet by the current performance of SCLIM2M in terms of spatiotemporal resolution in live cell imaging. It is not sufficient to be able to precisely track individual vesicles, even though we are much advanced than before. As shown in Figure 7B, moving average presentation may help trackability. Frame rates and pixel sizes of cameras are making rapid progress now and the methods of imaging-based single photon counting and restoration calculation can be further improved. Improvement of PSF determinism through optimization of the optical system will also be a stepping-stone to more rigorous restoration calculations. The number of photons observed is another limiting factor (photon budgets), but due to the rapid advancement in developing novel photon-emitting probes, we can also expect to handle more photons. Among the gene-encoded fluorescent markers, StayGold, recently developed by Miyawaki’s group (Hirano et al., 2022; Ando et al., 2024), is extremely photostable and will drastically change the world of fluorescent-protein-based live imaging.

## Data Availability

The original contributions presented in the study are included in the article/Supplementary Material, further inquiries can be directed to the corresponding author/s.

## Abbreviations

2D, 2 dimensional; 3D, 3 dimensional; 4D, 4 dimensional; CSU, confocal scanning unit; EMCCD, electron multiplying charge coupled device; epi, epifluorescence microscopy; I.I., image intensifier; MCP, microchannel plate; PSF, point spread function; ROI, region of interest; SCLIM, super-resolution confocal live imaging microscopy; sCMOS, scientific complementary metal oxide semiconductor; S/N, signal to noise ratio; TGN, *trans*-Golgi network.
