# Supplementary material for: Extremely high spatiotemporal resolution microscopy for live cell imaging by single photon counting, noise elimination, and a novel restoration algorithm based on probability calculation
Source: Front Cell Dev Biol. 2024 Jun 24;12:1324906. doi: 10.3389/fcell.2024.1324906 (PMC11228276; doi:10.3389/fcell.2024.1324906)
Supplement: Supplementary file 2 [file DataSheet1.pdf]

APPENDICES

Appendix 1. Noise-free single photon counting based on image processing

High-sensitivity measurement at a one-photon level is possible with commercially available cameras such as cooled EMCCD, cooled sCMOS and photomultiplier tubes, and their applications in photon counting modes are available. However, measurement at true one-photon accuracy is not easy, because the presence of noises often causes a problem. The image-processing single photon counting method employed in this study can remove all noises after the optical signal enhancement by I.I., thus realizing measurement almost at one-photon accuracy.

Supplemental Figure S1 shows an example of actual measurement. The image on the camera derived from a single photon is determined from the structure of the I.I., the pixels of the camera, and the magnification of the relay lens connecting them, and is represented by a convolution of a circular and Gaussian distribution (Equation 1). As shown in Supplementary Figure S1A, toward the raw image on the left, calculation was made to give a fitted model on the right. With this model, fitting is successful if individual single photons make well separate images on the camera at various concentrations (Supplementary Figure S1B). The optimal condition relies on finite resources in the entire measurement system and the accuracy of fitting depends on the number of valid photons counted.

$$Image_{single\ photon}(x) = Cir(r) * Gau(\sigma) * \delta(x)$$

Equation 1. The first term is circular function of radius *r*, the second term is a

Gaussian function of variance σ, and the third term is a delta function for position *x*. Convolution is hereafter denoted by *.

Appendix 2. Single photon counting method employed in this study and its advantage

As described in Appendix 1, it is optimal to set the measurement condition under which the concentration of photons is low enough to avoid overlapping. Under such conditions, the center pixel of a single-photon-derived image can be easily determined and the 8 pixels surrounding it are sufficient to distinguish the information due to the single photon from noises. This way enables computational processing at low load (Materials and Methods).

I.I. and high-speed camera are combined with a relay lens at variable magnification. Simulation calculations show that the optimum magnification is achieved when the density of camera pixels and that of MCP channels of I.I. are approximately equal. In the case of the combination of Photron FASTCAM camera and Hamamatsu I.I., which was used in live imaging measurements described in this paper, the camera pixel size (10 μm) is slightly larger than the pitch of I.I. MCP channels. If the magnification of the relay lens is appropriately adjusted, the amounts of light entering the central pixel and the eight peripheral pixels are optimally balanced. It should be noted here that, as I.I. takes a hexagonal dense structure and the camera has a square structure, camera pixels and MCP channels do not match perfectly. Its effect is also taken into account.

In addition to the regular noise components, which can be removed by the above calculations (Supplementary Figure S1B), very rare ion-feedback noises

occur at I.I. (Supplementary Figure S1C and D). They are recorded as an occasional very large, bright spot in the field of view. Such events are treated as pixels with a missing value by simply setting the value zero. Practically, this type of events was not problematic because their occurrence was very rare. In the cases they cannot be ignored, interpolation using a probability filter should be considered.

The single photon counting method used in the present study suppresses dark counts to 10^{-5}/MCP channel/s, at -25°C, and achieves one-photon accuracy with a quantum yield of 30% (from the catalogue spec of I.I.). The high temporal resolution (up to 1 ms) of measurement is another important characteristic of this method. For the comparison with other optical microscopy, Equation 2 gives the definition of S/N. Due to the high magnitude amplification by cooled I.I., the SCLIM2M system acquires a 10^6 times larger amount of information than conventional spinning-disk microscopy, which is a great advantage to improve the spatiotemporal resolution. The practically effective restoration method depends on the amount of information.

$$SNratio = \frac{\frac{S}{E}}{\sqrt{\frac{S}{E} + D}},$$

$$Information\ per\ unit\ time = SNratio \cdot \frac{dim}{T_{me}}$$

Equation 2. *S* is the signal amount, *E* is the minimum distinguishable amount, *D* is the dark noise, *dim* is the dimension of the instrument, and T_{me} is the measurement time.

The conventional deconvolution algorithm, which restores the spatial resolution using only the information within the transmission band of the optical system, provides relatively stable results even from a small amount of information (Agard et al., 1989). There, various noise models are assumed and their merits in estimation are discussed, but all of them are based on point estimation (a method of selecting one optimal image by some statistical criterion). This method does not give any measure of how reliable the chosen image is. If there is an infinite amount of information, point estimation can provide a reliable answer, but with a realistic finite amount of information, assuming out-of-band extrapolation, only an image with uncertain reliability (one that depends on random noise) can be obtained. In actual calculation processes, high-frequency noise components are simultaneously emphasized during iterative calculations for convergence or restoration of attenuated high-frequency signal components. To avoid such noise amplification, conventional deconvolution algorithms build in noise filters that cut high-frequency information (high-resolution part in space domain). This frequency cut was the major cause of the limitation of deconvolution algorithms in achieving super-resolution.

On the contrary, by the method developed in this paper, we can utilize the information of single photons with accurate x, y, z, and t values. As described below, we are now able to reconstruct original structures without special measures for noise elimination. To improve the spatial resolution to the extent extrapolatable outside the transmission bandwidth, we need a novel algorithm described in Appendix 3. It assumes that an extremely large amount of information is available, which has been realized in this study. In contrast to

conventional point estimation, this method corresponds to interval estimation, in which all estimated candidate images are considered according to statistical criteria.

Appendix 3. Restoration theory

The relationship between the original structure to be recovered (GI), the structure transmitted through the microscope system and formed on the observation plane (Im), and the distribution of photons observed (Ph) is represented by Equation 3. In general, restoration is performed by transforming between these pieces of information, but it is necessary to select a space (e.g., spatial resolution) for the restoration destination that is equivalent to the amount of information in the observed data (in this case, Ph). This model selection process corresponds to the process of choosing the trade-off between intensity information and spatial resolution for a given purpose in general optical systems.

$$PDF(Ph) = Im = PSF * GI$$

Equation 3. PDF is probability density function. PSF is point spread function.

In fluorescence microscopy, the question can be simplified to determination of the positions of fluorescent molecules. In a homogeneous population, the expected frequency of their photon emission is regarded to be constant for all molecules. This assumption is implicit in general fluorescence microscopy. In other words, obtaining a fluorescence microscopic image is simply drawing a map of the expected numbers of fluorescent molecules present in the observation

space. As a result, the amount of information can be much smaller to recover the original structure (GI).

Suppose that N photons are emitted and observed from M fluorescent molecules, where M is unknown and an undefined number from 1 to N is assumed. In this case, the distribution of expected values (GI') of M fluorescent molecules, which is estimated from the observed N photons (Ph), is expressed by Equation 4. The expected reconstructed structure (GI') is now expressed as integration of ∏ (product of sequences for N photons) of individual photon positions as convolved with PSF and GI. It can be further transformed to ∑ (summation) for M molecules. The restored images in this paper are calculated based on this formula. The homogeneity of the fluorophores discussed above can be included in the calculation as conditions related to M, resulting in improved resolution due to the reduction of the amount of information to be recovered.

$$GI' = \int GI \prod_{n}^{N} \delta n * PSF * GI$$

$$= \int \sum_{m}^{M} \delta m \prod_{n}^{N} \delta n * PSF * \sum_{m}^{M} \delta m$$

Equation 4. Expression of the distribution GI' given the measurements of N photons, assuming an unknown number of molecules M.

The main purpose to obtain the microscopic image GI' as above is to intuitively grasp the phenomenon observed. In some cases, such intuitive understanding can directly lead to a conclusion, but in other cases, a more

precise mathematical analysis may be necessary to conclude. The information obtained by the SCLIM2M system in this study can be treated in a more rigorous manner in probabilistics. Here, the problem of PSF, which is discussed in Appendix 4, is important. In this paper, we have successfully obtained precise and high spatiotemporal resolution images in a style of conventional fluorescence microscopy, which are valuable for intuitive discussion. Furthermore, the results obtained by this methodology provide mathematically more refined information. Because the measured value derives from the number of photons and its accuracy is guaranteed, the meaning of the estimated value (GI') is much more explicit. It can be used, for example, for estimating the number of molecules, determining shape, and finding correlations between the positions of multiple objects.

Another point to note is the inhomogeneity of the zt plane. The observation method of this study in the zt plane is based on the information obtained through a limited window of waveforms to estimate the rest. In analyzing the object motion more precisely, it is desirable to take into account the framework of the above rigorous probability theory. This time, we decided not to go further into the problem of motion, which is negligible during the short time of scanning in the cases we deal with. Nevertheless, the comparison of different time widths in moving averages, as shown in Figure 7, is primitive but turns out practical for considering motion.

Appendix 4. Handling of PSF

An attempt to determine the PSF of SCLIM2M was made by measurement of a

fluorescent bead as shown in Supplementary Figure S2. The result indicates that the apparent PSF is distorted in all directions. In particular, the characteristics along the optical axis (z axis) are not only asymmetric but also discontinuous and unrepresentable in elementary functions. Furthermore, as the actual observation of biological specimens involves spatial heterogeneity in the refractive index, it is difficult to accurately determine PSF at each location in the 3D field of view.

The PSF values used in the calculations of this study were based on the optics of a spinning disk confocal microscope. Designed PSF values are available, but they are often not appropriate in actual measurements (Kimura and Wilson, 1993). In this study, the measured values were used to correct the designed values. For the optical axis direction in particular, which contained irregular distortions as shown in Supplementary Figure S2, we tried to buffer the uncertainty by convolving a Gaussian distribution of appropriate magnitude based on the measured values. This led to underestimate of the optical performance. We are aware that the ambiguous treatment of PSF limits the accuracy of restoration calculation and therefore affects the spatial resolution. Its refinement should be addressed in the future.

Appendix 5. Supporting information of the measurement and calculation process

The numerical performance of the microscopic system SCLIM2M is summarized in Table 1.

The excitation light power is pre-measured on the actual sample surface, and the ND filter is adjusted in the range of 0.01-100% to suit the observation.

The excitation light intensity is designed to be controlled precisely, because the method is quantitative in principle and further high-precision mathematical analysis is expected. Since the amount of information obtained from a single fluorescent photon is dramatically improved by our original single photon counting, the excitation light intensity can be reduced by several orders of magnitude compared to other super-resolution microscopy methods under equivalent conditions. Thus, SCLIM2M is very resistant to bleaching. In our daily observations, about 200 3D images can be obtained almost without bleaching. The numbers of 3D images that can be obtained under similar conditions are several tens for SCLIM1 and about 5 for stimulated emission depletion microscopy.

Instrument resources are allocated according to the expected observations. Practically, the size of the data storage (on-chip memory of cameras) determines the number of consecutive images that can be captured, because large data transfer from the cameras to the computer takes a significant time. In the time domain, a trade-off exists between the temporal resolution and the time available for continuous observation. In addition, the temporal resolution is limited by the minimum exposure time of the camera, and the temporal resolution of the 3D image is limited by the scanning method in the z-direction. We are aware that these limitations on the range of imaging in the zt plane, including z-direction operations, are "time window problems," and we consider this an issue to be addressed in the future.
