# Supplementary material for: Extremely high spatiotemporal resolution microscopy for live cell imaging by single photon counting, noise elimination, and a novel restoration algorithm based on probability calculation
Source: Front Cell Dev Biol. 2024 Jun 24;12:1324906. doi: 10.3389/fcell.2024.1324906 (PMC11228276; doi:10.3389/fcell.2024.1324906)
Supplement: Supplementary file 5 [file Image1.pdf]

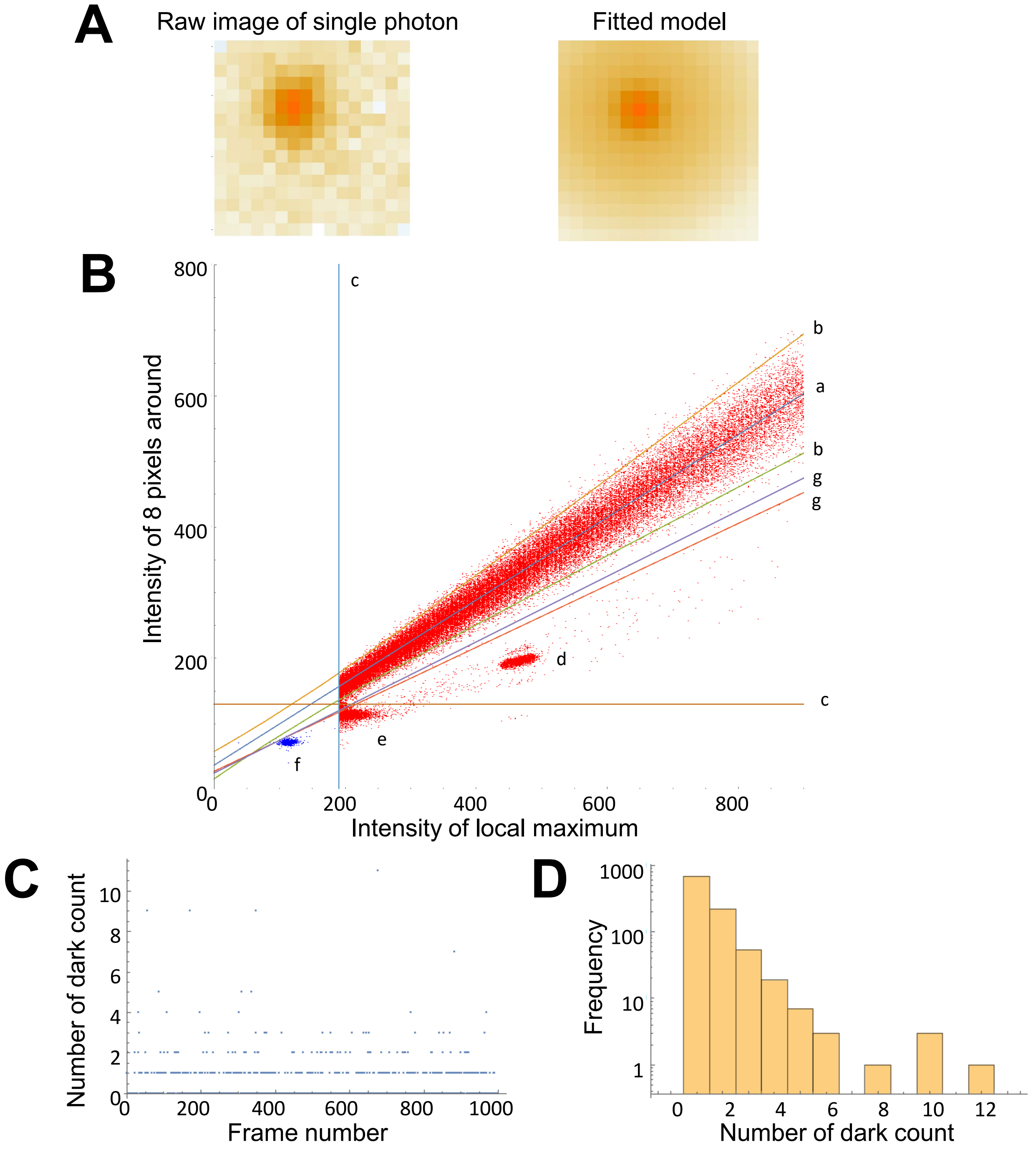

Figure S1. Single photon counting example

(A) A raw image derived from a single photon (left) and its fitted model (right). (B) Relationship between intensity values of the pixel at local maximum and those of 8 surrounding pixels. Line 'a' represents the ratio estimated from the model. Lines 'b' represent the upper and lower limits calculated from fluctuations in intensity values and positional relationship between MCP channels and camera pixels. Lines 'c' are set as the lower limits to exclude dark noises from the camera. Areas 'd' and 'e' represent noises derived from individual characteristics of cameras, which are excluded from the count by shape determination. They are generally recognized as known noises. Area 'f' represents dark noises that arise with the same shape as photon images and are excluded from the count by the intensity value. Lines 'g' represent the range where false counts are expected due to interference between camera-specific noises and photon images. (C) A result of counting for 1000 frames taken with the incident light level set to 0. (D) Histogram of C. The vertical axis is displayed in a logarithmic scale.

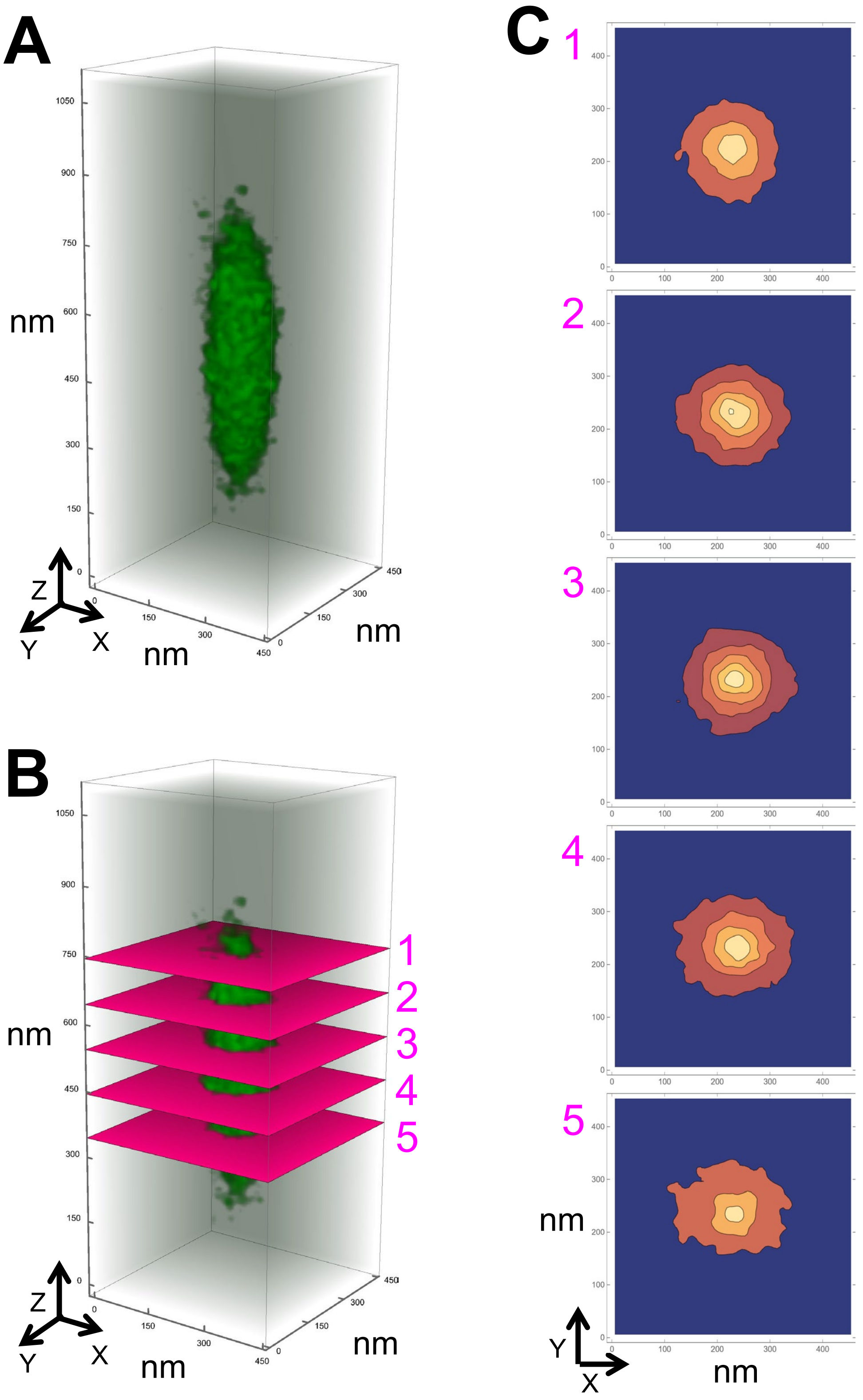

Figure S2. PSF measurement data
(A) A 3D opacity display of the observed image of a fluorescent bead. Data were obtained by single photon counting in the same way as other imaging experiments and the raw image without super-resolution restoration processing is shown. (B) To the 3D image of A, xy slices were positioned along the z-axis. (C) Tomogram at the positions indicated in B, with the intensity displayed as a contour map.

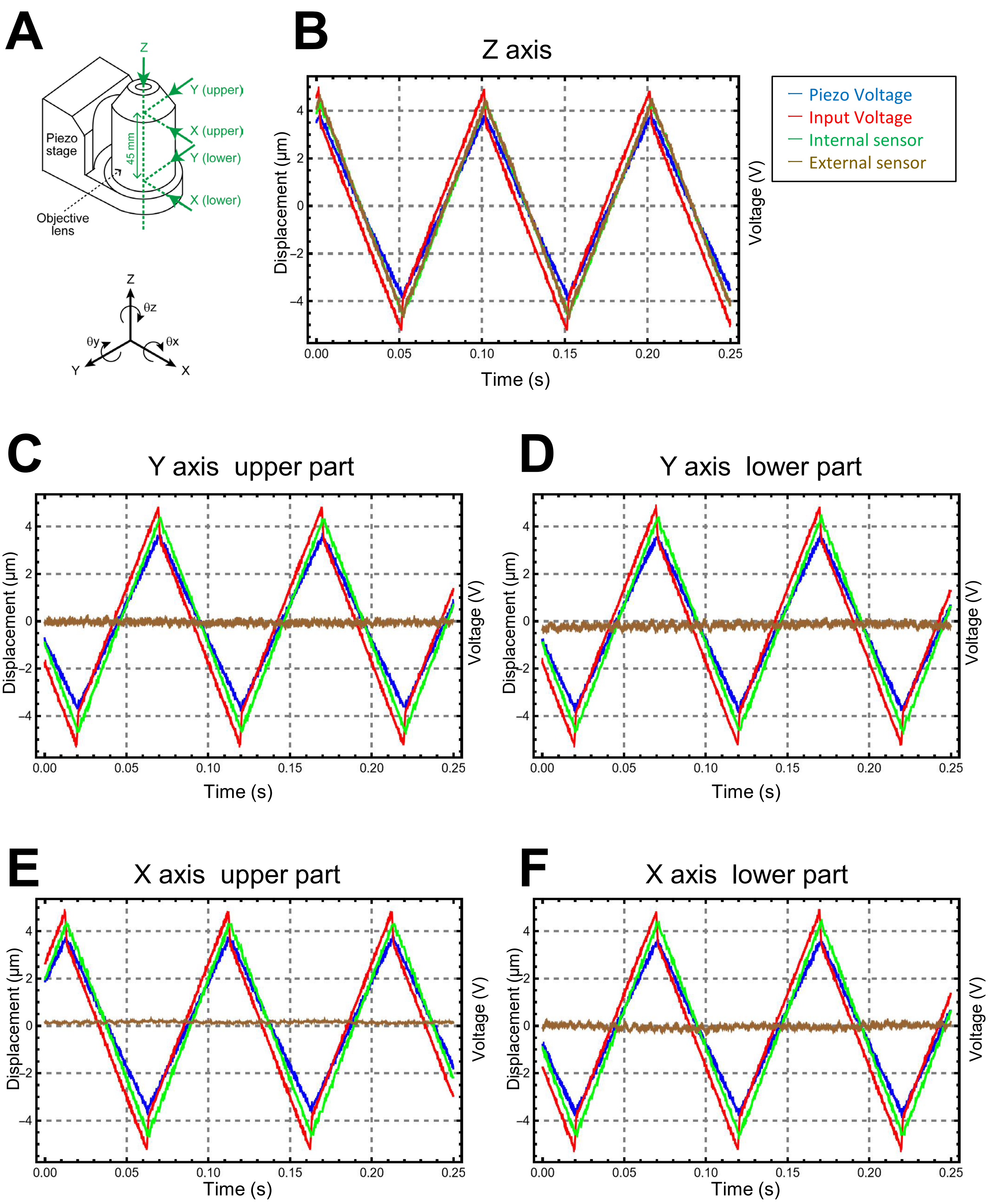

Miyashiro et al., Figure S3-1

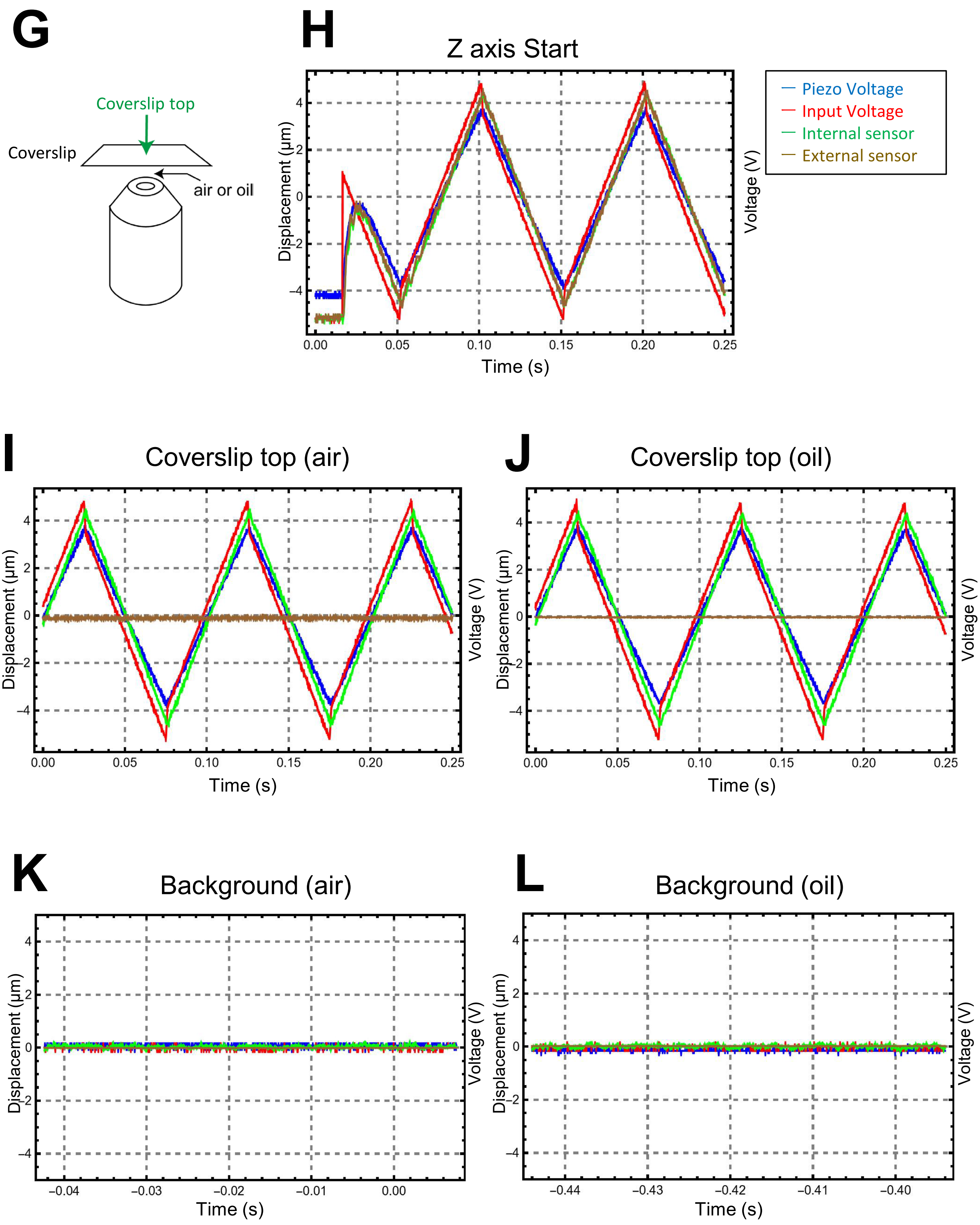

Miyashiro et al., Figure S3-2

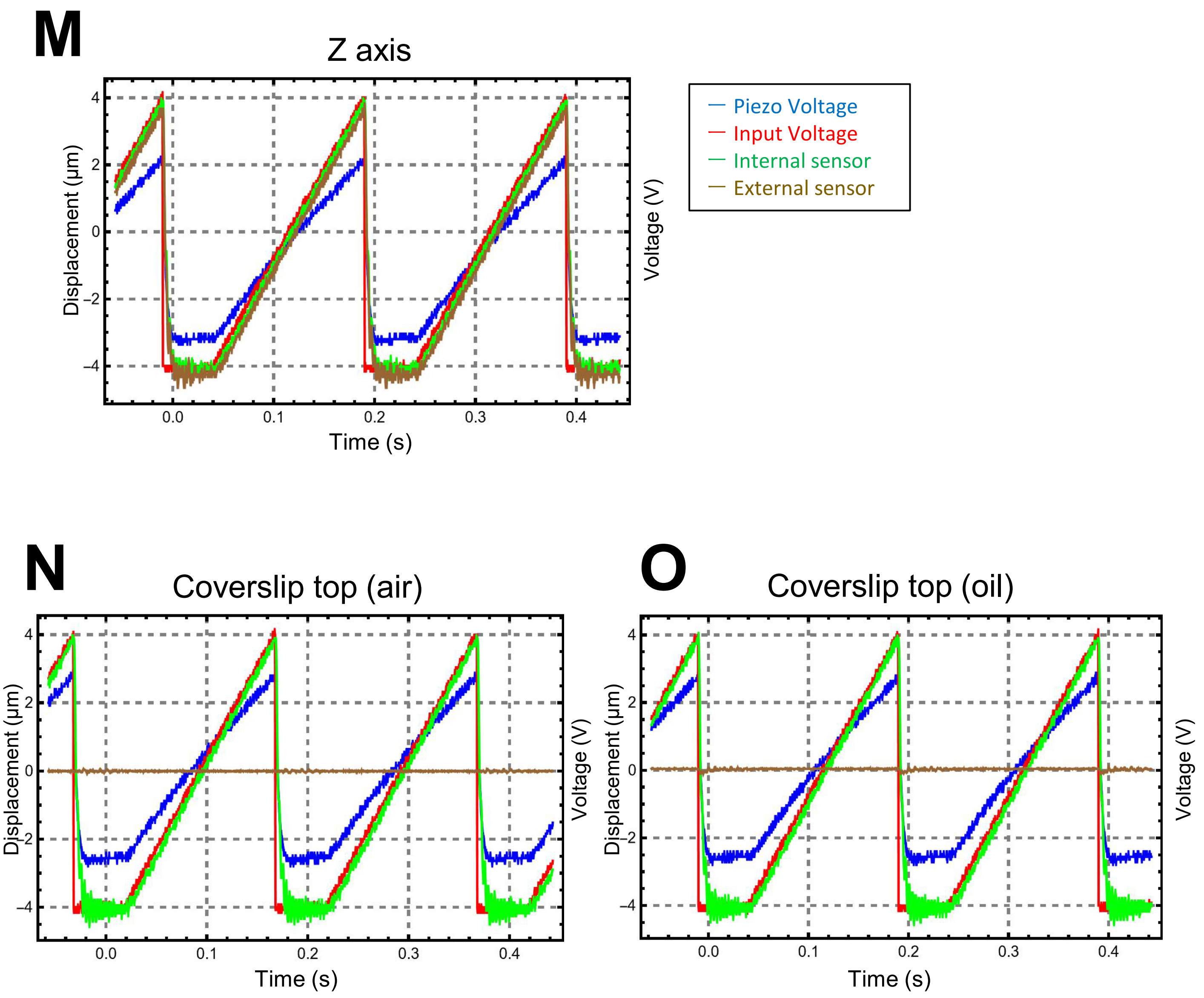

Figure S3. Measurements of piezo operation to test scanning accuracy
(A) Positional relationship between the objective lens, the piezo stage and optical axis directions. (B) A result on the relationship between the piezo-applied voltage, the input voltage, the piezo-stage internal sensor values and external sensor measurements in the triangular-wave mode. The internal sensor is used in the negative feedback loop of the piezo stage. The external sensors are used to measure the actual movement of the objective lens. (C-F) Results of measurements made for each position and direction shown in A. (G) The sensor arrangement used to measure movement of the observation surface. (H) Transient response at the start of triangular-mode oscillation. In actual data acquisition, imaging was started after the first two cycles to eliminate transient responses. (I-L) Results of the measurement for the configuration as shown in G. The background values of the lens in air and when immersed in oil are also shown. (M) Piezo operation in a saw-wave mode. (N and O) Results of the measurements with air and oil for the configuration of G during the saw-wave mode.

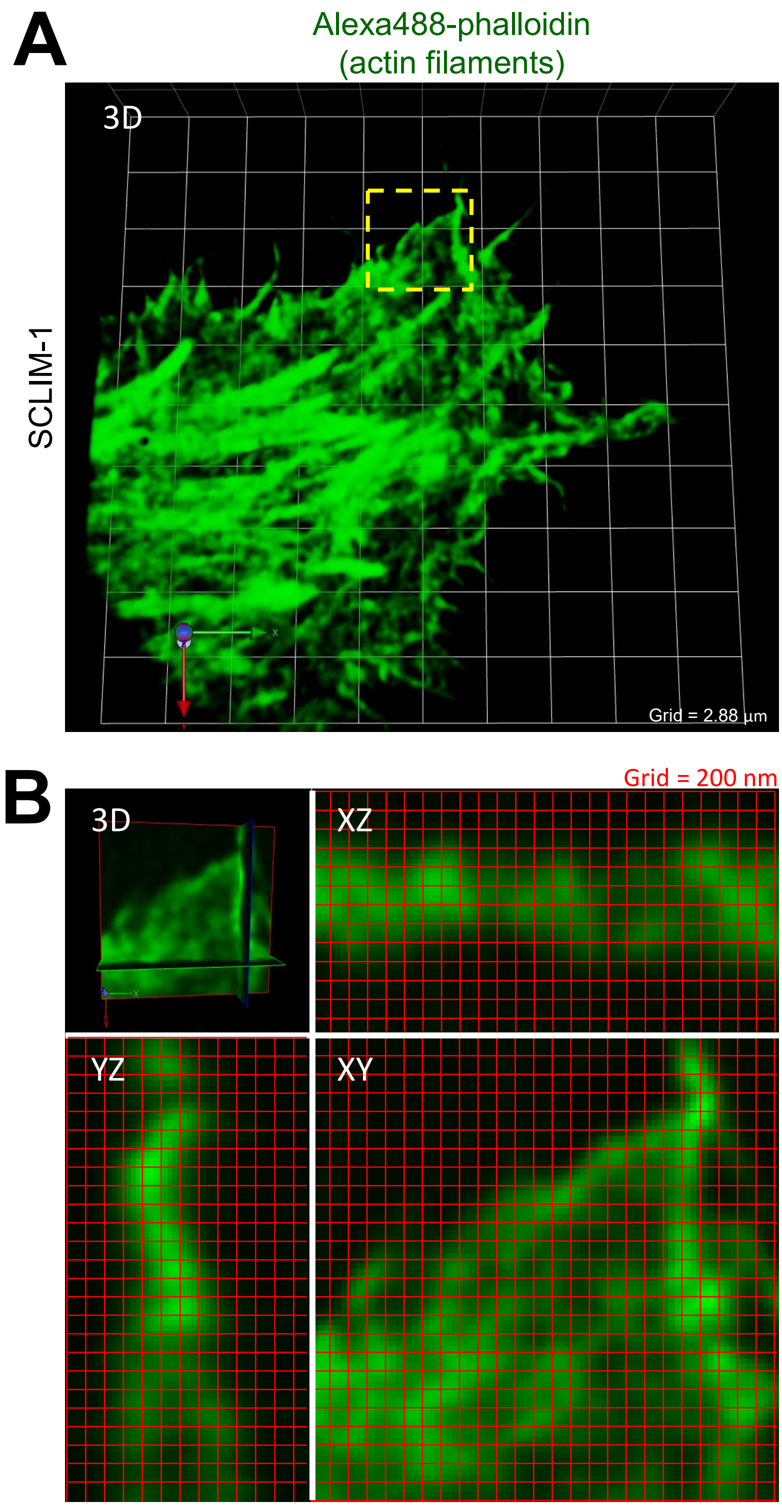

Figure S4. SCLIM1 observation of actin filaments in a fixed HeLa cell
Actin filaments in a chemically fixed HeLa cell were visualized by phalloidin staining. (A) A 3D image of the peripheral region of the cell taken by SCLIM1. (B) Zoom-up images of actin filaments in the yellow dashed box drawn in A. Images of single XY, XZ, and YZ sections are shown. Grid size, 200 nm.

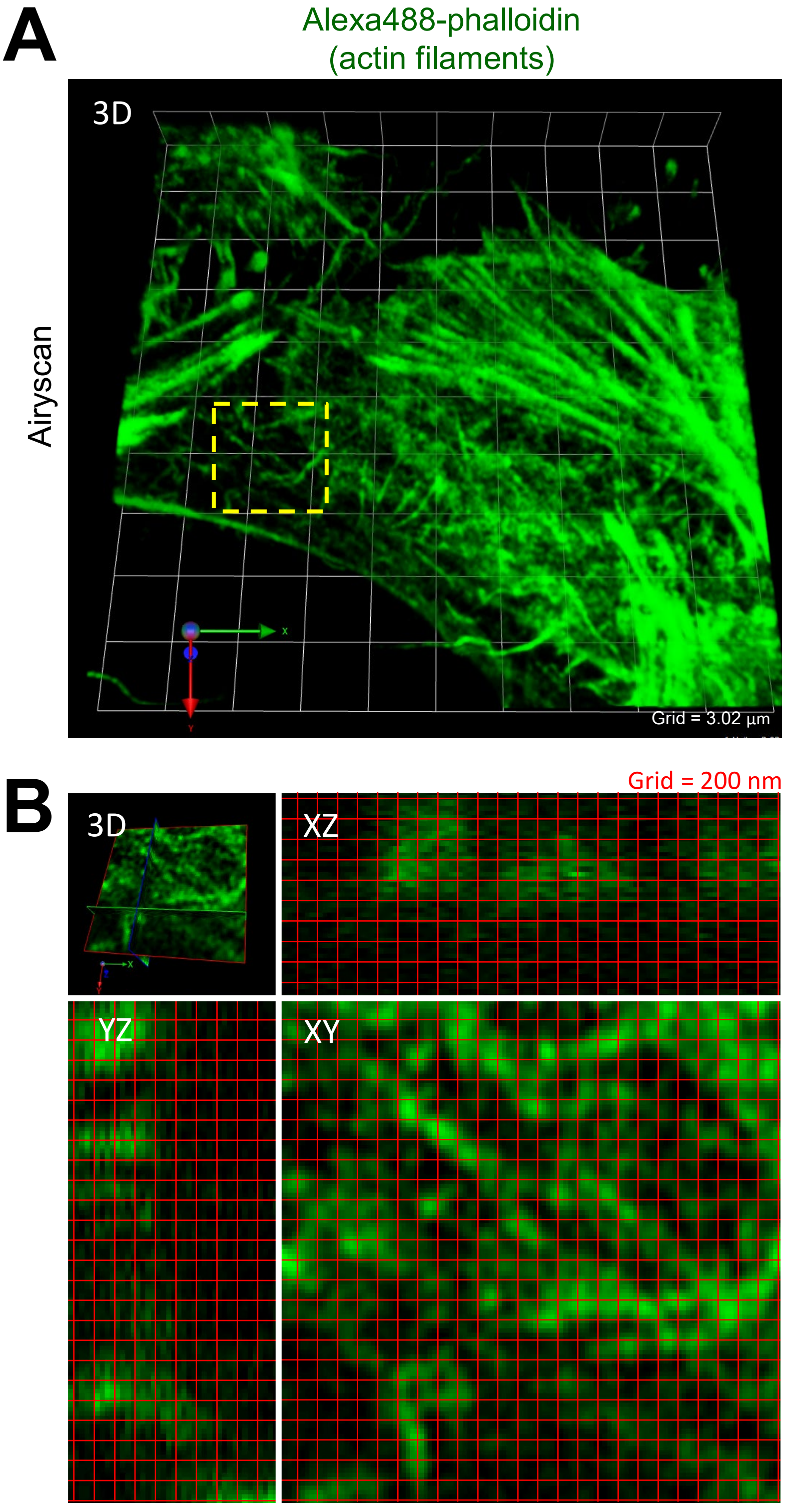

Figure S5. Airyscan observation of actin filaments in a fixed HeLa cell
Actin filaments in a chemically fixed HeLa cell were visualized by phalloidin staining. (A) A 3D image of the peripheral region of the cell taken by Zeiss LSM980 with Airyscan. (B) Zoom-up images of actin filaments in the yellow dashed box drawn in A. Images of single XY, XZ, and YZ sections are shown. Grid size, 200 nm.

SUPPLEMENTARY MOVIE LEGENDS

Movie S1. Dynamics of the Golgi apparatus in a living HeLa cell
Triple-color 4D movie of a portion of a living HeLa cell expressing mCherry-ManII (magenta, medial Golgi marker), iRFP-ST (cyan, *trans* Golgi marker), and mEmerald-TGN46 (green, TGN marker) (shown in Figure 6). The red numbers that appear in the upper right corner represent the time (s) after the onset of image acquisition. First, the still image of time zero is rotated to show 3D arrangement, and then the separation of three markers is shown. After the timer starts, 3D data obtained in every 150 ms separated by 50 ms are displayed as a movie at 5 volumes/s (real time).

Movie S2. Clathrin dynamics at the TGN in a living yeast cell
Dual-color 4D movie of a living yeast cell expressing Sec7-tagRFP (magenta, TGN marker) and Clc1-GFP (green, clathrin light chain) (shown in Figure 7). The red numbers in the upper right corner represent the time (s) after the onset of image acquisition. 3D data were obtained by a triangular-wave mode (Pattern 1, see Figure 7B) taken at every 50 ms (no blank) and compiled into the movie, which is played at 20 volumes/s (real time).

Movie S3. Dynamics of the Golgi apparatus in a living HeLa cell observed by SCLIM1
Triple-color 4D movie of a portion of a living HeLa cell expressing mCherry-ManII (magenta, medial Golgi marker), iRFP-ST (cyan, *trans* Golgi marker), and mEmerald-TGN46 (green, TGN marker), observed by SCLIM1. The red numbers that appear in the upper right corner represent the time (s) after the onset of image acquisition. First, the still image of time zero is rotated to show 3D arrangement, and then the separation of three markers is shown. After the timer starts, 3D images acquired consecutively every 5 s are displayed as a movie. The acquisition time for a single 3D stack is 1.733 s.
